# A cross-sectional study of the current status of psychological health and its correlation with academic performance in medical students: taking medical students in a medical university in China as examples

**DOI:** 10.3389/fpsyt.2025.1496248

**Published:** 2025-02-11

**Authors:** Han Chunhong, Ding Jingjing, He Huan, Zheng Peiyao, Zhang Xiaona, Yang Xiaowen, Zheng Aiming

**Affiliations:** ^1^ School of Education Science, Nanjing Normal University (ED.D), Nanjing, Jiangsu, China; ^2^ Department of Teaching Management, Nanjing Medical University, Nanjing, Jiangsu, China; ^3^ Medical Education Institution, Nanjing Medical University, Nanjing, Jiangsu, China; ^4^ Student Affairs Department, Nanjing Medical University, Nanjing, Jiangsu, China; ^5^ School of Public Health, University of Sydney, Sydney, NSW, Australia; ^6^ School of Stomatology, Nanjing Medical University, Nanjing, Jiangsu, China; ^7^ Library, Nanjing Medical University, Nanjing, Jiangsu, China; ^8^ Psychological Health Education and Counseling Center, Nanjing Medical University, Nanjing, Jiangsu, China

**Keywords:** medical students, current status of mental health, academic performance, depression, perceived stress scale

## Abstract

**Background:**

The psychological health problems are becoming increasingly prominent among medical students under the heavy academic stress and high-intensity clinical internships. This study examines the psychological health of medical students in a Chinese university and its impact on academic performance, aiming to inform targeted interventions.

**Methods:**

2022 undergraduate and postgraduates from an independent medical university in Eastern China were selected to score using the Patient Health Questionnaire-9 (PHQ-9), Generalized Anxiety Disorder-7(GAD-7), Insomnia Severity Index (ISI), Perceived Stress Scale (PSS), and Beck Scale for Suicide Ideation(BSSI); and the total grade point averages (GPAs) of 2022 undergraduates in the first and second academic years were determined to analyse the main psychological influencing factors.

**Results:**

The general survey of 2022 new medical students at admission showed that the various scores of postgraduates were higher than those of undergraduates. After two semesters, the percentage of students scored higher in unhealthy psychology indicators was increased in both postgraduates and undergraduates, but the differences between two groups were decreased. Analysis of the first and second-year academic performances of 2022 undergraduates revealed that assessment scores of PHQ-9, PSS, ISI, and BSSI had a correlation with the total GPA. However, no correlation was found between the GAD-7 values and the GPA. The PHQ-9 scores and major categories were identified as independent factors influencing the average GPA.

**Conclusion:**

The undergraduates face significant challenges in depression, anxiety, insomnia, and perceived stress, and these have negative effects on their academic performances; in particular, the depressive symptoms and perceived stress can significantly reduce their academic performances. In contrast, the psychological health statuses in the postgraduates tend to deteriorate as the semester progress.

## 1 Introduction

The importance of psychological health in medical students cannot be ignored, as it is directly related to the quality of medical education and the practice ability of medical professionals in the future. The psychological health is not only a key element in the professional competence of medical practitioners, but also the foundation for medical students to successfully complete their studies and achieve career development. As the backbone of the future medical field, their psychological health statuses directly affect the quality and efficiency of medical services. Therefore, a deep understanding and concern about the psychological health statuses of medical students are of indispensable significance in cultivating medical talents with a good psychological quality. The long-term nature of medical education and high-intensity learning pressure make the psychological health problems particularly prominent in medical students, which urgently require in-depth research and attention from the academic community.

At present, some studies have shown that the academic stress, competitive learning environment, and challenges of clinical internships lead to the fact that the psychological health problems of medical students become more and more prominent. These problems not only seriously affect the quality of life and psychological health in medical students, but also may significantly reduce their academic performances, which in turn affect their future careers (1–4).

Further studies have shown that the medical students commonly face psychological health problems such as anxiety, depression and burnout. For example, in Italy, 8.6% of medical students reported experiencing psychological health problems during their studies, mainly including anxiety, depression and burnout (1). In the Portuguese-speaking region, more than 50% of medical students reported experiencing varying degrees of psychological health problems (2). A study in Egypt showed that 16% of medical students are diagnosed with a psychological illness during their studies, and a burnout test indicated that 88% of medical students reach a critical point of burnout (5). In addition, a study in Swiss showed that the prevalence of substance abuse among medical students is strongly associated with poorer psychological health status (6).

Gender differences, major public health events, and cultural diversity play key roles in the psychological health status in medical students. Some studies have revealed that the female medical students are more likely to suffer from anxiety and depression and show greater vulnerability in coping with burnout and stress compared to their male counterparts (2, 3, 7, 8). In addition, major public health events, such as COVID-19 pandemic, not only exacerbate the psychological burden of medical students, but also cause additional anxiety and depression in the transition to new learning modes (9–11). Psychological challenges faced by medical students in different countries and regions are also affected by their different cultural backgrounds and social support systems, which play a particularly significant role in high-pressure learning environments (4, 12).

In recent years, the mental health of Chinese medical students has gradually received attention from the academic community. Several studies have shown that medical students generally face high psychological pressure, especially due to the academic burden, the tension of clinical internships, and the uncertainty of their future careers, which have led to significantly higher mental health problems such as anxiety, depression, and insomnia among medical students than among the groups of college students of other specialties (13, 14). It has been found that the mental health status of medical students is affected by various factors, including the tension in the doctor-patient relationship, family expectations, economic pressure, and insufficient social support (15, 16). In addition, academic interest and professional identity are key factors affecting mental health. Those students who lack interest in the medical profession have more significant psychological problems such as anxiety, depression, and low self-esteem (17, 18). When coping with stress, medical students of different grades showed differences, with lower grades feeling more restless, while higher grades showed more burnout and insomnia (14, 18). In the context of big data analysis, the researchers also found that the research hotspots of medical students’ mental health focused on stress management and mental health education. With the outbreak of global public health events, such as the COVID-19 epidemic, the mental health problems of medical students have become more prominent, especially when they show higher risks of anxiety and depression when dealing with emergencies and long hours of intense work (19).

The negative correlation between psychological health problems and academic performance has also been widely validated. Some studies have found that symptoms of anxiety and depression can lead to a significant decrease in academic performances of medical students (3, 20). In addition, the psychological health problems can also trigger burnout and procrastination in medical students during their studies, further weakening their academic performances (21, 22). The academic burnout is characterized by the lack of learning interest and motivation, and the reduced sense of academic achievement. It has been found that academic burnout is significantly and negatively correlated with academic self-efficacy, professional identity, and social support. Positive emotions can reduce the academic burnout, while negative emotions can exacerbate the academic burnout (23–26).

There is a close link between the mental health of Chinese medical students and their academic performance, and studies have shown that mental health problems have a significant negative impact on academic performance. First, high levels of psychological stress and anxiety faced by medical students significantly affect their academic performance. One study showed that students with high psychological stress are more likely to experience burnout and decreased grades (27, 28). This stress stems not only from the academic burden, but also from family expectations, financial pressures, and challenges in clinical practice (29). For medical students, problems such as anxiety, depression, and insomnia can lead to lack of concentration and decreased learning efficiency, which ultimately affects their test scores and overall academic performance (30). Second, studies have shown that medical students with poor mental health have significantly lower academic performance than students with good mental health. For example, anxiety symptoms are significantly negatively correlated with academic performance, and the more severe the perceived stress and depressive symptoms, the worse the academic performance (27, 29, 30). These problems not only affect students’ classroom performance, but also their long-term career development (28). In addition, professional identity is also an important factor affecting the psychological health and academic performance of medical students. Studies have shown that students who lack interest or identity in medicine are more likely to have psychological problems, which in turn affect their academic performance (17). Therefore, increasing students’ interest and confidence in medicine and cultivating a positive sense of professional identity can help relieve their psychological stress and improve their academic performance (31).

Currently, international and Chinese studies on medical students’ mental health and its relationship with academic performance have progressed but are still deficient. First, most studies lack long-term tracking of the mental health status of medical students, and are unable to understand the dynamic process of change and the long-term impact of their mental health. Second, although some studies have pointed out that medical students generally face problems such as anxiety, depression, and burnout, most of them focus on a single point in time and lack comparative analysis of different stages of study, especially in the early stage of enrolment and the subsequent stage of study, the psychological changes of which are insufficiently researched. In addition, in China, studies on the mental health of medical students are relatively fragmented, with limited sample sizes and a lack of comprehensive comparisons between undergraduate and graduate student groups.

Given the limitations of these existing studies, this study selected 2022 new undergraduate and postgraduates at a medical university in China as subjects, comprehensively analysed their psychological assessment results and grade point averages (GPA) at the initial stage of their enrolment and two years later, thus providing a strong scientific basis for understanding and supporting the psychological health in medical students worldwide.

Research question 1: To assess the psychological health status of Chinese medical students, with a specific focus on their profiles across five critical dimensions: depression, anxiety, insomnia, perceived stress, and suicidal ideation. Additionally, to examine how these dimensions dynamically evolve over different stages of their academic careers (undergraduate versus postgraduate levels).

Research Question 2: To investigate the specific relationship between the psychological health status of Chinese medical students and their academic performance, assessing the impact of various psychological health indicators—such as depression, anxiety, insomnia, perceived stress, and suicidal ideation—on GPA. Additionally, to analyse the differential predictive roles of these indicators on academic outcomes.

## 2 Methodology

### 2.1 Assessment tools

A variety of standardized psychological assessment tools were used in this study to comprehensively assess the psychological health status in medical students. These include the Patient Health Questionnaire-9 (PHQ-9), the Generalized Anxiety Disorder-7(GAD-7), the Insomnia Severity Index (ISI), the Perceived Stress Scale (PSS), and the Beck Scale for Suicide Ideation (BSSI). Each of these scales is widely used in psychological health assessment with good reliability and validity.

#### 2.1.1 Patient health questionnaire-9

PHQ-9 is a self-assessment tool commonly used to screen for depression, it contains nine items, each of which corresponds to the frequency of a depressive symptom, with scores ranging from 0 to 27 (32). PHQ-9 is capable of not only assessing the severity of depressive symptoms, but also identifying mild, moderate, and severe depression through different score nodes. It is widely used in clinical and research settings, with good internal consistency and validity. The PHQ-9 has been widely validated for its applicability among elderly and general populations in China, demonstrating good reliability and validity. Research shows that the PHQ-9 is effective for depression screening in Chinese medical students and the general population, with proven cross-cultural applicability (33).

#### 2.1.2 Generalized anxiety disorder-7

GAD-7 is an instrument for the assessment of generalized anxiety disorder, and it consists of seven items, each of which is used to assess the frequency of anxiety symptoms in the past two weeks (34). The scores range from 0 to 21, and the higher the score is, the more severe the anxiety symptoms are. GAD-7 has a high degree of internal consistency and is commonly used in research and clinical practice to assess the presence of anxiety symptoms and their severities. The GAD-7 has been validated as suitable for anxiety assessment among Chinese patients. Multiple studies have confirmed its good internal consistency and construct validity in outpatient and hospital populations, supporting its widespread use within the Chinese cultural context (35).

#### 2.1.3 Insomnia severity index

ISI is a tool used to assess insomnia symptoms, which consists of 7 items that evaluate the severity of insomnia symptoms such as difficulty falling asleep, difficulty staying asleep, and early waking (36). The scores range from 0 to 28 points, and a higher score indicates more severe insomnia symptoms. ISI has been widely used in researches on insomnia, with good reliability and validity. The Chinese version of the Insomnia Severity Index (ISI) has shown high reliability and validity in studies of insomnia in Chinese adults, and can be used to assess the degree of insomnia in Chinese populations (37).

#### 2.1.4 Perceived stress scale

PSS is a scale that assesses the subjectively perceived stress in individuals, it contains 10 items (38). PSS is used to assess the subjective perception of life stress in an individual over the past month, and a higher score indicate a greater perceived stress. It has been shown that PSS has good reliability and validity across different populations and is a commonly used tool for stress assessment. The Chinese version of the PSS has undergone psychometric validation among Chinese police and other populations, demonstrating high reliability and validity, making it suitable for measuring subjective stress perceptions in Chinese populations (39).

#### 2.1.5 Beck scale for suicide ideation

BSSI is a tool used to assess the suicidal ideation in individuals, it contains 21 items, each of which assesses a specific cognition or behaviour related to suicidal ideation (40, 41). BSSI can effectively distinguish individuals at risk of suicide and assess the severity of their suicidal ideation. The scores range from 0 to 100 points, and a higher score indicates greater suicidal ideation. This scale is widely used in research and clinical screening for suicide risk. The BSSI has been widely used for assessing suicidal ideation among Chinese populations and has been validated for cross-cultural applicability, showing strong performance, particularly in measuring mental health-related issues (42, 43).

The test values for reliability and validity of all the above-mentioned scales were 0.84 for Cronbach’s α coefficient, 0.84 for the coefficient of Kaiser-Meyer-Olkin (KMO), and P < 0.001 for Bartlett’s test of sphericity, respectively, and the combined application of these scales can provide reliable data support for a comprehensive understanding of the psychological health status in medical students.

### 2.2 Research sample and assessment process

This study selected a prestigious independent medical university locating in a developed region in Eastern China, which is ranked among the top 2024 Soft Science Ranking of Chinese Universities and attracts students from different regions, ensuring that the study samples were diverse and representative. We conducted a comprehensive study of all 2022 new undergraduate and postgraduates, involving a total of 2799 undergraduates and 2,834 postgraduates from various medical majors such as basic medicine, and clinical medicine, thus providing diverse and rich data samples for this study.

In this study, a online questionnaire survey was conducted within a concentrated time period to assess the psychological health status in 2022 new undergraduate and postgraduates. The online assessment platform ensured the convenience and coverage of data collection and coverage, and all students could complete the questionnaire survey within a specified time frame via their personal devices.

#### 2.2.1 First assessment

The first assessment was conducted in new medical students at admission, this was designed to provide a comprehensive assessment of the initial psychological health status in new undergraduate and postgraduates. Measurements at this stage were taken for all freshmen, achieving full coverage of the freshman population and ensuring the reliability of the baseline data.

#### 2.2.2 Second assessment

The second assessment was scheduled at the end of the second semester with the aim of monitoring changes in the psychological health status in medical students as they adjusted to university life. A stratified sampling method based on variables such as discipline and gender was used in the second assessment of students to obtain representative sample data.

postgraduates who revealed a tendency to develop psychological problems in the first assessment would receive additional attention, and were further subject to continuous individual tracking and assessment in the second assessment for a more in-depth understanding of the dynamic changes and development trend in their psychological statuses. This two stage assessment approaches could effectively capture the changes in psychological health in medical students in the process of adapting to study and life, thus providing rich supporting data for subsequent analyses.

In the second psychological survey, we established a 50% sampling target for undergraduates, structured by four main academic categories. Stratified sampling was employed within each category, using convenience sampling to ensure representation across all majors. For graduate students, the sampling process proved more challenging, as many had transitioned to hospital or laboratory settings with their supervisors after completing the primary coursework in the first semester. Thus, a 15% sampling target was set for graduate students, mirroring the four-category stratified sampling approach used for undergraduates, with convenience sampling applied within each category. However, the level of cooperation and completion varied significantly across the graduate categories. Ultimately, the second-round participation rate was 46% for undergraduates and 12% for graduate students, both meeting our expected sampling proportions.

### 2.3 Acquisition of academic performance data

GPA was used an indicator for assessing the academic performances of undergraduates, and the scores on core and non-core courses of each undergraduate student were used as a statistical basis, and its calculation method was shown in the following formula. This indicator is a commonly used indicator for comparing the academic performance among medical students in different majors. In the logistic regression analysis, all undergraduates were divided into two groups according to GPA (2.93 ± 0.01 points): the students with a score greater than or equal to 2.93 points were set as the effective learning group, and those with a score less than 2.93 points were set as the non-effective learning group.


$$\text{GPA}=\frac{\sum_{\text{n}=1}^{\infty }\left(\text{Course assessment score}\times \text{Required course credit}\right)}{\sum_{\text{n}=1}^{\infty }\left(\text{ Required course credit}\right)}$$

### 2.4 Data analysis methods

In this study, the data analyses were conducted using a variety of statistical methods to comprehensively assess the relationship between the psychological health status and academic performance in the medical students. Various categorical variables such as gender and the grade corresponding to each scale score were compared using the Chi-square test; various continuous variables were expressed as the mean ± standard error, the paired t-test was performed (e.g., the differences in psychological scale scores in undergraduates were compared between two assessments), and the Pearson’s correlation analysis was performed(e.g., GPA and specific score on each scale), *P*<0.05 was used as the criterion for statistical significance.

A note on age: according to the selection system of higher education in China, the undergraduates in general medical colleges and universities are fresh graduates from senior high schools, and all of them are 19 years old at the time of admission; however, the postgraduates (master’s and doctoral students) must obtain a bachelor’s degree before they can start their studies, so their age is usually 5 years older than that of undergraduates, and no further comparisons between ages were conducted.

When the factors affecting the total GPA were assessed, and both univariate and multivariate logistic regression analyses were performed in this study. The variables with P<0.1 in univariate logistic regression analysis were included in the multivariate logistic regression analysis to ensure that no potentially influential factors were mistakenly excluded, while the multivariate analysis was performed to control the potential confounding factors and identify the key factors that could independently affect GPA, and *P*<0.05 was still used as the criterion for statistical significance to ensure that the results were reliable and scientifically valid. The combined application of these statistical methods contributed to a deeper understanding of the effects of psychological health statuses on their academic performances.

## 3 Results

### 3.1 Basic information of 2022 medical students and the first assessment scores

A total of 2,831 undergraduate and 2,834 graduate students were newly enrolled in this medical university in 2022. A gender ratio analysis indicated that the male-female ratio in the undergraduates was similar, while the proportion of females significantly exceeded that of males in the postgraduates, and this difference was statistically significant.

PHQ-9 was used for the psychological health assessment, in which, 0-4 points indicated no depression (category I), 5-9 points indicated mild depression (category II), 10-14 points indicated moderate depression (category III), 15-19 points indicated moderately severe depression (category IV), and 20 points and above indicated severe depression (category V). The results showed that the percentage of students without depression was lower in the undergraduates than in the postgraduates, while all the percentages of students with mild to severe depression were higher in the undergraduates than in the postgraduates, as detailed in **Table 1**.

**Table 1:** Psychological scores of 2022 new medical students at admission (autumn 2022).

Item		Undergraduates	Postgraduates	Reference statistic value(t/χ^2^)	P
		n1 = 2799	n2 = 2834		
Age		18.10 ± 0.02	23.39 ± 0.05	100.60^◊^	<0.001^*^
Gender	Female	1471(52.50%)	1729(61.01%)	41.40^▴^	<0.001^*^
	Male	1330(47.50%)	1105(38.99%)		
PHQ-9	I	2355(84.10%)	2463(86.90%)	13.05^▴^	<0.001^*^
	II	373(13.30%)	330(11.60%)		
	III	51(1.80%)	31(1.10%)		
	IV-V	20(0.80%)	10(0.40%)		
GAD-7	I	2526(90.20%)	2616(92.30%)	8.77^▴^	<0.001^*^
	II	235(8.40%)	195(6.90%)		
	III-IV	38(1.40%)	23(0.80%)		
ISI	I	2626(93.80%)	2704(95.40%)	7.04^▴^	0.004^*^
	II	159(5.70%)	120(4.20%)		
	III-IV	14(0.50%)	10(0.30%)		
PSS	I	1046(37.40%)	1177(41.50%)	14.51^▴^	0.001^*^
	II	1505(53.80%)	1459(51.50%)		
	III-IV	248(8.80%)	198(7.00%)		
BSSI	I	2479(88.6%)	2767(97.60%)	182.32^▴^	<0.001^*^
	II	268(9.60%)	61(2.20%)		
	III	52(1.90%)	6(0.20%)		

^*^:significant difference between two groups.^◊^:t test. ^▴^:χ^2^ test.

The scoring criteria for GAD-7 was as follows: 0-4 points indicated no anxiety (category I), 5-9 points indicated mild anxiety (category II), 10-14 points indicted moderate anxiety (category III), and 15-21 points indicated severe anxiety (category IV). The percentage of students without anxiety was similarly lower in the undergraduates than in the postgraduates, whereas the percentages of students with mild to severe anxiety were higher in the undergraduates than in the postgraduates, see details in **Table 1**.

The scoring criteria for ISI were as follows: 0-7 points indicated no insomnia (category I), 8-14 points indicated mild insomnia (category II), 15-21 points indicated moderate insomnia (category III), and 22-28 points indicated severe insomnia (category IV). The percentage of students who did not report insomnia was lower in the undergraduates than in the postgraduates, while the percentages of students with mild to severe insomnia were higher in the undergraduates than in the postgraduates, and see details in **Table 1**.

The scoring criteria for PSS were as follows: 0-13 points indicated no stress (category I), 14-28 points indicated mild stress (category II), 29-42 points indicated moderate stress (category III), and 43-56 points indicated severe stress (category IV). A lower percentage of undergraduates reported no stress than the postgraduates, while higher percentages of undergraduates reported mild to severe stress than postgraduates, as shown in **Table 1**.

BSSI had a score range of 0-100 points, the students with a score of 60 points and above were marked as a group that requires special attention. The percentage of students with a score of more than 60 points was significantly higher in the undergraduates than in the postgraduates (p<0.0001), which is reflected in **Table 1**.

### 3.2 Basic information of 2022 medical students and the second assessment scores

In this study, a questionnaire survey was still conducted in 2022 undergraduates of this medical university in April 2023 using a general survey method to ensure the comprehensiveness and representativeness of data sources as much as possible. A stratified random sampling survey was conducted in 2022 postgraduates, since they had completed their courses in the first semester, and most of them had already begun doing some researches under the guidance of their mentors and no longer lived in the school dormitory.

A total of 1326 undergraduate and 347 graduate students finally completed the assessment in this survey. The male-female ratio among undergraduates was roughly equal, and the proportion of females significantly exceeded that of males in the postgraduates, and this difference was statistically significant. This comparison revealed that there were no longer differences in GAD-7, ISI and PSS scores between the two groups, and the percentage of students with higher ISI scores was increased in both undergraduate and postgraduates, with the increase being more pronounced in postgraduates. The statuses corresponding to the scoring categories of PHQ-9, GAD-7, PSS, and BSSI were better in postgraduates than in undergraduates, and the differences were still statistically significant, which is reflected in **Table 2**.

**Table 2:** Psychological scores of 2022 new medical students at the second assessment (spring 2023).

Item		Undergraduate students	Postgraduate students	Reference statistic value(t/χ^2^)	P
		n1=1324	n2=347		
Age		18.47±0.02	23.64±0.15	60.45^◊^	<0.001^*^
Gender	Female	776 (58.69%)	241 (69.45%)	13.61^▴^	<0.001^*^
	Male	548 (41.31%)	106 (30.55%)		
PHQ-9	I	1003 (75.76%)	287 (82.70%)	7.79^▴^	0.02^*^
	II	271 (20.47%)	50 (14.41%)		
	III-V	50 (3.77%)	10 (2.89%)		
GAD-7	I	1124 (84.89%)	306 (88.18%)	3.05^▴^	0.22
	II	168 (12.69%)	36 (10.37%)		
	III-IV	32 (2.42%)	5 (1.45%)		
ISI	I	1207 (91.16%)	322 (92.80%)	1.35^▴^	0.51
	II	105 (7.93%)	23 (6.63%)		
	III-IV	12 (0.91%)	2 (0.57%)		
PSS	I	417 (33.79%)	122 (35.16%)	1.83^▴^	0.40
	II	768 (58.01%)	192 (55.33%)		
	III-IV	139 (10.50%)	33 (9.51%)		
BSSI	I	1287 (97.21%)	326 (93.95%)	9.75^▴^	0.01^*^
	II	31 (2.34%)	20 (5.76%)		
	III	6 (0.45%)	1 (0.29%)		

^*^: significant difference between two groups. ^◊^: t test. ^▴^: χ^2^ test.

### 3.3 Comparative analysis of index changes in 2022 undergraduates between the two assessments

This study conducted a before-after comparative analysis of 1,326 undergraduates who participated in the two assessments in autumn 2022 and spring 2023, respectively. The results showed that all other psychometric index scores except the BSSI score showed an increasing trend as the academic year progressed, and this change was statistically significant. Specifically, all scores on the dimensions of anxiety, depression, insomnia, and stress in undergraduates were increased, reflecting an increase in their psychological burden as the learning tasks became more demanding. However, it was worth noting that there was a significant decrease in BSSI score, which may indicate an improvement in suicidal ideation or progress in coping with extreme emotional distress in students, as shown in **Table 3**.

**Table 3:** Paired t-test for two measured values of various indexes in 2022 undergraduates.

Index	N	Mean ± SE		t	P
		1^st^-time Investigation	2^nd^-time Investigation		
PHQ-9	1323	1.72 ± 0.08	2.53 ± 0.11	-7.91	<0.001^*^
GAD-7	1323	1.13 ± 0.07	1.58 ± 0.08	-5.54	<0.001^*^
ISI	1323	1.94 ± 0.08	2.28 ± 0.10	-3.34	0.001^*^
PSS	1323	32.06 ± 0.27	33.87 ± 0.26	-6.29	<0.001^*^
BSSI	1323	6.78 ± 0.43	2.87 ± 0.25	8.81	<0.001^*^

^*^:significant difference between two groups.

### 3.4 Correlation and regression analyses between the two assessment scores and academic performances in 2022 undergraduates

This content was investigated in the second part of the study, which delved into the correlation between the two assessment scores and academic performances in 2022 undergraduates. The total GPA at the end of the second semester was used as a quantitative index of academic performance to analyse the potential effects of psychological health status on academic performances, and detailed correlation and regression analyses were conducted.

As shown in **Table 4**, depressive symptoms, perceived stress and suicidal ideation had a significant negative effect on the academic performance of medical students, while the anxiety symptoms and insomnia had a relatively small or insignificant effect on the academic performance of medical students.

**Table 4:** Correlation analysis between total GPA and various indexes.

	PHQ-9-1	PHQ-9-2	GAD-7-1	GAD-7-2	ISI-1	ISI-2	PSS-1	PSS-2	BSSI-1	BSSI-2	Total GPA
PHQ-9-1	1										
PHQ-9-2	0.41<0.001*	1									
GAD-7-1	0.69<0.001*	0.41<0.001*	1								
GAD-7-2	0.38<0.001*	0.76<0.001*	0.47<0.001*	1							
ISI-1	0.58<0.001*	0.34<0.001*	0.51<0.001*	0.34<0.001*	1						
ISI-2	0.31<0.001*	0.67<0.001*	0.33<0.001*	0.67<0.001*	0.36<0.001*	1					
PSS-1	0.41<0.001*	0.28<0.001*	0.43<0.001*	0.27<0.001*	0.35<0.001*	0.19<0.001*	1				
PSS-2	0.25<0.001*	0.37<0.001*	0.24<0.001*	0.40<0.001*	0.21<0.001*	0.32<0.001*	0.43<0.001*	1			
BSSI-1	0.38<0.001*	0.17<0.001*	0.35<0.001*	0.18<0.001*	0.33<0.001*	0.15<0.001*	0.21<0.001*	0.09 0.001*	1		
BSSI-2	0.22<0.001*	0.33<0.001*	0.24<0.001*	0.24<0.001*	0.17<0.001*	0.29<0.001*	0.16<0.001*	0.19<0.001*	0.24<0.001*	1	
Total GPA	-0.04 0.14	-0.07 0.02*	0.01 0.95	-0.01 0.85	-0.04 0.18	-0.02 0.51	-0.05<0.001*	-0.15<0.001*	0.02 0.52	-0.08 0.002*	1

The total GPA of 2022 undergraduates was 2.94 points, whereby 2022 undergraduate student were divided into two groups: the group with a score above or equal to total GPA, and the group with a score below total GPA. Thereafter, the univariate and multivariate logistic regressive analyses on the factors that may affect the total GPA were conducted, and the potentially influential variables were screened by using the univariate logistic regressive analysis, as shown in **Table 5**.

^*^:significant difference between two groups.

Correlation between PHQ-9 scores and academic performances: two measured PHQ-9 (PHQ9-1 and PHQ9-2) scores were significantly and negatively correlated with the total GPA (r1=-0.04, *P*=0.02; r2=-0.07, *P*=0.02). This implies that the more severe the depressive symptoms are, the worse the academic performances of medical students are.

Correlation between PSS scores and academic performances: two measured PSS (PSS-1 and PSS-2) scores were significantly and negatively correlated with total GPA (r1=-0.11, *P*<0.001; r2=-0.15, *P*<0.001). This indicates that the higher the perceived stress is, the worse the academic performance is. This result suggests that the perceived stress has a significant negative effect on the academic performance in medical students.

Correlation between ISI scores and academic performances: there was a significant negative correlation between the firstly measured ISI(ISI-1) score and total GPA (r=-0.05, *P*=0.01), while there was no significant correlation between the secondarily measured ISI(ISI-2) score and total GPA. This may indicate that the insomnia has an effect on academic performance in the early stages of study, but this effect may diminish or be masked by other factors over time.

Correlation between BSSI scores and academic performances: there was a significant negative correlation between the secondarily measured BSSI(BSSI-2) score and total GPA (r=-0.08, P=0.002), whereas there was no significant correlation between the firstly measured BSSI (BSSI-1) score and total GPA. This suggests that increased suicidal ideation may have a more significant negative effect on academic performance at the end of the semester.

Correlation between GAD-7 scores and academic performances: two measured GAD-7 scores were not significantly correlated with total GPA(both p>0.05),suggesting that the effects of anxiety symptoms on academic performance may be more limited or less pronounced.

The data in **Table 5** indicated that the depressive symptoms, perceived stress and suicidal ideation were important factors affecting the academic performances of medical students. Among them, the depressive symptoms and suicidal ideation had a significant negative effect on academic performance, especially at the end of the semester. The anxiety and insomnia, although common psychological problems, did not show a significant effect on academic performance in this study.

**Table 5:** Univariate logistic regressive analysis of factors affecting learning outcomes of 2022 undergraduates.

Variable	β	S.E.	Wald	P	Exp(β)	95%CI
Gender^▴^	-0.48	0.11	17.72	<0.001*	0.62	0.49-0.78
PHQ-9-1^▴^	-0.04	0.02	5.34	0.02*	0.96	0.92-0.99
PHQ-9-2^▴^	-0.03	0.01	5.83	<0.02*	0.97	0.93-0.99
GAD-7-1^▴^	-0.004	0.02	0.03	0.87	1.00	0.96-1.05
GAD-7-2^▴^	-0.02	0.02	1.31	0.25	0.98	0.94-1.02
ISI-1^▴^	-0.03	0.02	2.83	0.09*	0.97	0.93-1.00
ISI-2^▴^	-0.18	0.02	1.38	0.24	0.98	0.95-1.01
PSS-1^▴^	-0.02	0.006	7.08	0.008*	0.98	0.97-0.99
PSS-2^▴^	-0.09	0.007	10.36	0.001*	0.98	0.97-0.99
BSSI-1^▴^	0.004	0.004	1.58	0.21	1.00	0.99-1.01
BSSI-2^▴^	-0.02	0.006	5.91	<0.02*	0.98	0.97-0.99
Majors^▴^			115.16	<0.001*		
Major category 1	0.55	0.30	3.52	0.06	1.74	1.08-2.35
Major category 2	-0.57	0.31	2.65	0.10	0.60	0.33-1.11
Major category 3	-0.83	0.30	7.48	0.006*	0.44	0.24-0.79

Analysis of majors was performed by taking the clinical major as the benchmark category, major category 1: clinical auxiliary specialty such as nursing, medical laboratory technology, medical imaging technology; major category 2: basic disciplines and specialties such as basic medicine, pharmacy, and preventive medicine; major category 3: medical-related liberal arts majors such as English, public utilities management, and medical insurance. ^▴^: dependent variable. *: P<0.1 as significant difference between two groups.

PHQ-9: Two measured PHQ-9 scores were significantly correlated with academic performances (PHQ-9-1: P=0.02, Exp[β]=0.83; PHQ-9-2: P<0.01, Exp[β]=0.79), indicating that the more severe the depressive symptoms are, the poorer the academic performances are. In particular, the second measured score (i.e., the end of the semester) could more accurately predict a decline in academic performance.

PSS: the firstly measured PSS score was significantly and negatively correlated with the academic performance (p<0.01, Exp[β]=0.83), but the secondarily measured PSS score showed a correlation with the academic performance only at marginal levels of significance (p=0.09). This may indicate that perceived stress has a greater effect on the academic performances of medical students in the early stages of their studies, and the medical students may gradually adapt to stress as the semester progresses.

BSSI: the secondarily measured BSSI score was significantly correlated with the academic performance (p<0.01, Exp[β]=0.66), but the firstly measured BSSI score did not reach the significance level. This may reflect the fact that as the semester progresses, the negative effect of suicidal ideation on academic performance becomes more apparent, especially when faced with academic challenges and stress.

GAD-7 and ISI: both the two measured GAD-7 and ISI scores showed no significant effects on the academic performance(all > 0.05). This suggests that although the anxiety and insomnia are common psychological health problems, their direct effects on academic performance may be limited or these effects may be masked by other factors.

A multivariate logistic regression analysis was conducted on the basis of the above variables and the detailed multivariate analysis results are presented in **Table 6**.

**Table 6:** multivariate logistic regression analysis of factors affecting academic performance of 2022 undergraduates.

Variable	β	S.E.	Wald	P	Exp(β)	95%CI
Gender^▴^	-0.79	0.13	40.07	<0.001^*^	0.45	0.35-0.58
PHQ-9-2^▴^	-0.03	0.02	3.95	0.04^*^	0.97	0.94-0.99
Majors^▴^			132.01	<0.001^*^		
Major category 1	0.86	0.30	8.07	0.005^*^	2.36	1.31-4.29
Major category 2	-0.31	0.32	0.93	0.34	0.74	0.40-1.37
Major category 3	-0.73	0.31	5.71	0.02^*^	0.48	0.26-0.88

Analysis of majors was performed by taking the clinical major as the benchmark category, major category 1: clinical auxiliary specialty such as nursing, medical laboratory technology, medical imaging technology; major category 2: basic disciplines and specialties such as basic medicine, pharmacy, and preventive medicine; major category 3: medical-related liberal arts majors such as English, public utilities management, and medical insurance. ^▴^:dependent variable. ^*^:significant difference.

The results of multivariate regression analysis in **Table 6** indicated that depressive symptoms and major categories were independent factors affecting the academic performances of medical students. Among them, the severity of depressive symptoms had a negative effect on academic performance, while the students in different majors showed significant differences in academic performances.

PHQ-9: the secondarily measured PHQ-9 (PHQ9-2) were significantly and negatively correlated with the academic performance (p<0.01, Exp[β]=0.78), indicating that the more severe the depressive symptoms are, the worse the academic performances of medical students are. This implies that depressive symptoms have a greater effect on academic performances at the end of the semester and may affect the earning efficiency and attentional concentration in medical students.

Majors: the major categories also showed a significant effect in the regression analysis (p<0.001). In particular, major category 2 (basic disciplines and specialties such as basic medicine, pharmacy, and preventive medicine) was significantly positively correlated with the academic performance (P<0.001, Exp[β]=2.27), whereas major category 3 (medicine-related liberal arts majors such as English, public utilities management, and medical insurance) was significantly negatively correlated with the academic performance (P=0.02, Exp[β]=0.67). This may reflect that the content and difficulty of studies in different majors have different effects on the academic performances of medical students.

The scores (psychological load) for the first major category (Category One: Clinical Auxiliary Fields, including Nursing, Medical Laboratory Technology, Medical Imaging Technology, etc.; Category Two: Basic Science Fields, including Basic Medicine, Pharmacy, Preventive Medicine, etc.; Category Three: Humanities-Related Medical Fields, including English, Public Administration, and Medical Insurance) were relatively low. This result is associated with factors such as stable career aspirations and clear goals. Rankings in various indicators varied across other major categories, with the second major category showing the highest psychological load across multiple scores, as shown in **Supplementary Figure 1**.

Therefore, in this study, there was no interaction effect of PSS and ISS on GPA. Similarly, we analysed the interaction effects of eight other pairs of combinations—PHQ-9 and GAD-7, PHQ-9 and ISI, PHQ-9 and PSS, PHQ-9 and BSSI, GAD-7 and ISI, GAD-7 and BSSI, ISI and BSSI, and PSS and BSSI. The results indicated no interaction effect on academic performance for any of these pairs.

## 4 Discussion

### 4.1 Current psychological health status of medical students

This study conducted two surveys on the psychological health of 2022 undergraduate and postgraduates to reveal the changes in their psychological health at different stages of their studies. The findings showed that both undergraduate and postgraduates had significant psychological health problems during their studies, especially in terms of anxiety, depression, insomnia and perceived stress. It is noteworthy that in the first assessment, the psychological health status in postgraduates was significantly better than that in undergraduates. However, as the semester progressed, the psychological health status in postgraduates deteriorated; although it was still superior to that in undergraduates, the gap in psychological health status between the two groups of students narrowed significantly. Although the postgraduates might have a relatively stable psychological state due to richer life experiences and higher psychological maturity at the time of admission, their psychological burdens also significantly increased as the research progressed and the academic stress increased. This is consistent with the results of the study by El-Gabry et al., which noted that the psychological health of medical students gradually deteriorates during their senior years due to increased academic stress and workload in the clinical internship (8, 44, 45).

Two assessments in this study showed that the undergraduates had significantly higher levels of depression, anxiety, insomnia, and perceived stress than postgraduates. This result may reflect the adaptation problems faced by undergraduates during the transition from high school to university, as well as the high-intensity studying requirements of the medical majors themselves. In contrast, the postgraduates may be better equipped to deal with these challenges due to their previous educational experiences and life experiences. However, it is worth noting that although the postgraduates had better initial psychological health, their psychological health deteriorated more rapidly in the second assessment, especially in terms of insomnia and perceived stress. This may be related to the increased responsibilities of postgraduates in academic research and clinical internship. The study by Quek, T et al. indicated that compared with their junior counterparts, the senior medical students and postgraduates are more prone to experience deterioration in their psychological health during clinical internships and research due to work-related stress and time management issues (46).

Research indicates that undergraduate and graduate students at a particular Chinese medical university face significant mental health challenges, particularly in terms of depression, anxiety, insomnia, and perceived stress. This aligns closely with Wang J.’s findings, which report prevalence rates of depression and anxiety among Chinese medical students at 27% and 32%, respectively, primarily attributed to academic pressures and uncertainties regarding future career development (27). Additionally, similar to findings from a study conducted in Shandong Province (47), this research also reveals a progressive decline in mental health status across academic years, with pronounced increases in anxiety and depression, particularly during clinical internship stages.

### 4.2 Correlation between psychological health and academic performance

This study found that there was a significant negative correlation between depressive symptoms and academic performances of medical students. This is in line with the findings of the study on medical students by Dyrbye et al. in the United States, which showed that depressive symptoms can significantly weaken the learning efficiency and academic performance of medical students (48). A previous study has also pointed out that the depressive mood has a significant effect on the learning state of students, leading to a decline in their academic performances (49). Research by Chinese scholars similarly indicates that depression symptoms are prevalent among Chinese medical students, largely due to academic pressure and uncertainties surrounding future career prospects. The negative impact of depressive moods on academic performance is particularly pronounced. For instance, a study by Wang et al. reveals a high prevalence of depression among Chinese medical students, significantly impairing their focus and academic achievement (27, 47). These results emphasize the importance of early identification and intervention of depressive symptoms to avoid their long-term negative effects on the academic and career development of students.

This study showed that the perceived stress was significantly and negatively correlated with the academic performance, which is in line with the findings of many studies. The study by Saraswathi et al. revealed that the medical students tend to show poor academic performance and are prone to experience academic burnout when faced with a high level perceived stress (20). Research by Chinese scholars also indicates that perceived stress impacts the academic performance of medical students. Zheng et al. found that in high-pressure environments, medical students are more prone to academic burnout, with perceived stress notably increasing during examination periods and clinical internships, which negatively affects their motivation and concentration (27, 47). This result suggests that perceived stress management is critical to the academic success of medical students, and the schools should provide effective stress management strategies, especially during the most stressful period such as examination and internship.

This study also found that an increase in suicidal ideation was significantly correlated with a decrease in academic performance. A study by Dyrbye et al. also pointed out that the suicidal ideation is closely correlated with the academic burnout and emotional distress in medical students (48). The suicidal ideation is not uncommon among medical students, which is closely correlated with the academic stress and future career uncertainty they face (50). Research by Chinese scholars indicates that suicidal ideation among Chinese medical students is influenced by a combination of academic pressure, societal expectations, and uncertainties regarding career development (27, 47). These findings suggest that the schools should not only pay attention to the academic performance of students, but also establish an effective crisis intervention mechanism to identify and help students at risk of suicide in a timely manner.

Although the direct effects of anxiety and insomnia on academic performance were not significant in this study, this does not mean that these problems do not have an effect on quality of life and long-term academic performance in students. The study by Moutinho, I. L. D., et al. and Zheng (47) revealed that although the short-term effects of anxiety symptoms may be minimal, the failure to effectively manage anxiety and insomnia can gradually erode the learning motivation and academic achievements of students in the long term (51).

Suicidal ideation, insomnia, anxiety, and stress are symptomatic expressions of depression (52, 53). This study reveals a correlation between these symptoms and depression scores, aligning with the conclusions drawn by Yin and Zhang (52, 53). However, the primary finding of this research indicates that the PHQ-9 score, which serves as a depression metric, is a critical factor influencing the academic performance of medical students, while psychological burdens reflected by instruments such as the GAD-7 do not exhibit the same level of impact.

The rationale for this observation lies in the fact that students with depression often experience varying degrees of the “three low symptoms” associated with the disorder—namely, diminished mood, slowed cognitive processing, and reduced volitional activity. These symptoms contribute to a decline in cognitive capabilities, which can adversely affect attention and memory. Consequently, depression has a direct effect on academic performance, whereas anxiety and other psychological burdens do not directly influence students’ cognitive functioning.

In summary, this study posits that the depression indicator (PHQ-9) demonstrates an independent correlation with academic achievement, thereby establishing its potential as an effective psychological predictor and intervention reference for educational performance.

Therefore, in order to improve the psychological health status of medical students and enhance their academic performances, the medical universities and colleges can establish an education model integrating medical treatment and prevention, which can be performed through the synchronized implementation of five combinations (combination of medicine with education, home-school partnership, functional integration,cooperation between universities and hospitals, and integrating postgraduate and undergraduate education) and the reasonable application of five methods of psychological education (forward-looking planning, integrated guidance, precise intervention, interactive assistance and all-round escort); a joint conference mechanism should be set up for the promotion of psychological health education, and a general psychological test can be carried out based on a big data platform for screening of psychiatric disorder, so as to conduct hierarchical and classified management of medical students. Some psychological counselling centres and “one-stop” student communities should be set up, and a model integrating five educations(moral,physical,aesthetic and labour educations) and psychological health education can be implemented to enrich the forms of psychological health education. Systematic training and training for class psychological counsellors should be carried out to enhance their professional literacy, and attention is paid to theoretical research, and typical case sets are compiled to promote a virtuous cycle between psychological health and academic development in medical students. This research and project will be further conducted in relevant universities of our medical education academic community to better promote the medical education.

The specific implementation methods include the following three aspects: First, since 2020, a mandatory 2-credit mental health course has been offered, requiring participation from all incoming undergraduates. Second, the university promotes a “five-in-one” educational approach, with the psychology department emphasizing the combined benefits of psychological and physical well-being. Third, regular group counselling sessions are held, along with individual counselling for students identified as at-risk during initial screenings, with approximately 16 sessions offered per week. Additionally, the university prioritizes communication with students’ parents, creating a “home-school partnership.” For students with more severe issues, timely referrals are made to professional mental health facilities, forming a “university-clinic collaboration” to provide continued specialized care.

## 5 Conclusion

This study reveals the changes in the psychological health of medical students at different stages of study and their correlations with academic performances by dynamically tracking the psychological health statuses of 2022 undergraduates and postgraduates in a medical university in China. It is found that both undergraduates and postgraduates face significant psychological health challenges in terms of depression, anxiety, insomnia and perceived stress, and these issues tend to worsen as the semester progresses. In particular, the depressive symptoms and perceived stress have a more significant negative effect on academic performance among undergraduates. The novelty of this study lies in systematically analysing the changes in psychological health of medical students in China in their academic progress and the effects of these changes on academic performance through combined use of cross-sectional and dynamic longitudinal studies. Initially, postgraduates exhibited better psychological health than undergraduates, likely due to their richer life experiences and higher psychological maturity. However, as the semester progressed, the psychological health of postgraduates deteriorated more rapidly, particularly in terms of insomnia and perceived stress. This aligns with findings that senior medical students and postgraduates are more prone to psychological health deterioration due to increased academic and research pressures.

In contrast, undergraduates faced higher levels of depression, anxiety, insomnia, and perceived stress throughout the study period. This may reflect the adaptation challenges they encounter transitioning from high school to university, as well as the demanding nature of medical studies. Despite the initial advantage of postgraduates, the gap in psychological health status between the two groups narrowed significantly as the semester progressed.

The findings provide an important scientific basis for developing more precise and sustained psychological health interventions in medical universities and colleges in the future. By identifying and understanding the similarities and differences in psychological health between undergraduate and postgraduates, this study offers new insights and recommendations for improving the total psychological health of medical students as well as enhancing their academic performances.

### 5.1 Actionable implications

#### 5.1.1 Early identification and intervention

Given the significant negative impact of depressive symptoms on academic performance, early identification and intervention are critical. Universities should implement regular mental health screenings to identify students at risk and provide timely support.

#### 5.1.2 Stress management programs

The strong correlation between perceived stress and academic performance suggests the need for stress management programs. These could include workshops, mindfulness training, and counselling services to help students cope with academic and personal stressors.

#### 5.1.3 Integrated education model

The study recommends an integrated education model that combines medical treatment and prevention. This can be achieved through the synchronized implementation of five combinations (*medicine with education*, *home-school partnership*, *functional integration*, *university-hospital cooperation*, and *integrating postgraduate and undergraduate education*) and the application of five methods of psychological education (*forward-looking planning*, *integrated guidance*, *precise intervention*, *interactive assistance*, and *all-round escort*).

#### 5.1.4 Crisis intervention mechanisms

The increase in suicidal ideation and its negative impact on academic performance highlights the need for effective crisis intervention mechanisms. Universities should establish protocols for identifying and supporting students at risk of suicide.

#### 5.1.5 Enhanced psychological counseling services

The findings suggest that psychological counselling centres and “one-stop” student communities should be established to provide comprehensive support. Systematic training for class psychological counsellors is also recommended to enhance their professional literacy.

### 5.2 Research gaps and future directions

#### 5.2.1 Broader variable assessment

Future studies should include a broader range of variables that may influence academic performance, such as social support, extracurricular activities, and financial stress, to provide a more comprehensive understanding of the factors affecting medical students’ mental health and academic performance.

#### 5.2.2 Longitudinal studies

The cross-sectional design of this study limits the ability to establish causation. Longitudinal studies are needed to track the psychological health and academic performance of medical students over multiple years, providing insights into the long-term effects of interventions.

#### 5.2.3 Multi-institutional studies

Expanding the sample to include multiple universities would enhance the generalizability of the findings. Multi-institutional studies can provide a more balanced and widely applicable perspective on the mental health challenges faced by medical students.

#### 5.2.4 Cultural and regional differences

Further research should explore the impact of cultural and regional differences on the psychological health of medical students. This can help tailor interventions to specific contexts and populations.

By addressing these actionable implications and research gaps, future studies and interventions can better support the psychological health and academic success of medical students, ultimately contributing to the development of a more resilient and effective medical workforce.

## 6 Limitations

This study has certain limitations. First, the absence of assessment for additional variables that may influence academic performance may limit the generalizability of the findings. Including a broader range of relevant factors would contribute to a more comprehensive understanding of influences on academic outcomes. Second, the sample was confined to a single university and employed a cross-sectional design, which restricts the ability to establish causation. Future studies addressing these aspects could provide a more balanced and widely applicable perspective on the results.

## Data Availability

The raw data supporting the conclusions of this article will be made available by the authors, without undue reservation.
