# Supplementary figures and images for: A cross-sectional study of the current status of psychological health and its correlation with academic performance in medical students: taking medical students in a medical university in China as examples
Source: Front Psychiatry. 2025 Feb 11;16:1496248. doi: 10.3389/fpsyt.2025.1496248 (PMC11850339; doi:10.3389/fpsyt.2025.1496248)

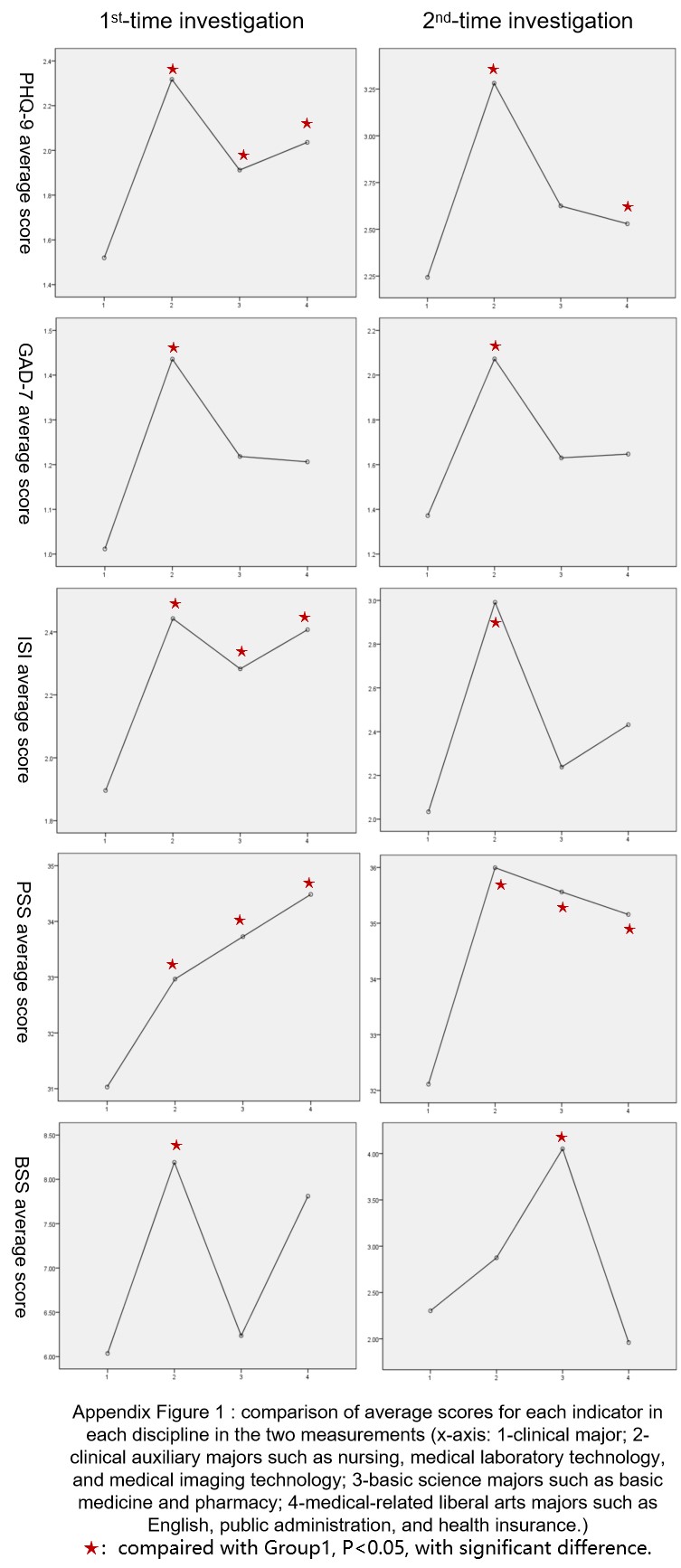

Supplement: Supplementary file 1 [file SupplementaryFile1.docx]
